# Docking Characterization and *in vitro* Inhibitory Activity of Flavan-3-ols and Dimeric Proanthocyanidins Against the Main Protease Activity of SARS-Cov-2

**DOI:** 10.3389/fpls.2020.601316

**Published:** 2020-11-30

**Authors:** Yue Zhu, De-Yu Xie

**Affiliations:** Department of Plant and Microbial Biology, North Carolina State University, Raleigh, NC, United States

**Keywords:** flavan-3-ols, flavan-3-ol gallates, procyanidins, COVID-19, green tea, muscadine grape, cacao, dark chocolate

## Abstract

We report to use the main protease (M^pro^) of SARS-Cov-2 to screen plant flavan-3-ols and proanthocyanidins. Twelve compounds, (–)-afzelechin (AF), (–)-epiafzelechin (EAF), (+)-catechin (CA), (–)-epicatechin (EC), (+)-gallocatechin (GC), (–)-epigallocatechin (EGC), (+)-catechin-3-O-gallate (CAG), (–)-epicatechin-3-O-gallate (ECG), (–)-gallocatechin-3-O-gallate (GCG), (–)-epigallocatechin-3-O-gallate (EGCG), procyanidin A2 (PA2), and procyanidin B2 (PB2), were selected for docking simulation. The resulting data predicted that all 12 metabolites could bind to M^pro^. The affinity scores of PA2 and PB2 were predicted to be −9.2, followed by ECG, GCG, EGCG, and CAG, −8.3 to −8.7, and then six flavan-3-ol aglycones, −7.0 to −7.7. Docking characterization predicted that these compounds bound to three or four subsites (S1, S1′, S2, and S4) in the binding pocket of M^pro^ via different spatial ways and various formation of one to four hydrogen bonds. *In vitro* analysis with 10 available compounds showed that CAG, ECG, GCG, EGCG, and PB2 inhibited the M^pro^ activity with an IC_50_ value, 2.98 ± 0.21, 5.21 ± 0.5, 6.38 ± 0.5, 7.51 ± 0.21, and 75.3 ± 1.29 μM, respectively, while CA, EC, EGC, GC, and PA2 did not have inhibitory activities. To further substantiate the inhibitory activities, extracts prepared from green tea (GT), two muscadine grapes (MG), cacao, and dark chocolate (DC), which are rich in CAG, ECG, GAG, EGCG, or/and PB2, were used for inhibitory assay. The resulting data showed that GT, two MG, cacao, and DC extracts inhibited the M^pro^ activity with an IC_50_ value, 2.84 ± 0.25, 29.54 ± 0.41, 29.93 ± 0.83, 153.3 ± 47.3, and 256.39 ± 66.3 μg/ml, respectively. These findings indicate that on the one hand, the structural features of flavan-3-ols are closely associated with the affinity scores; on the other hand, the galloylation and oligomeric types of flavan-3-ols are critical in creating the inhibitory activity against the M^pro^ activity.

## Introduction

**Figure 1:**
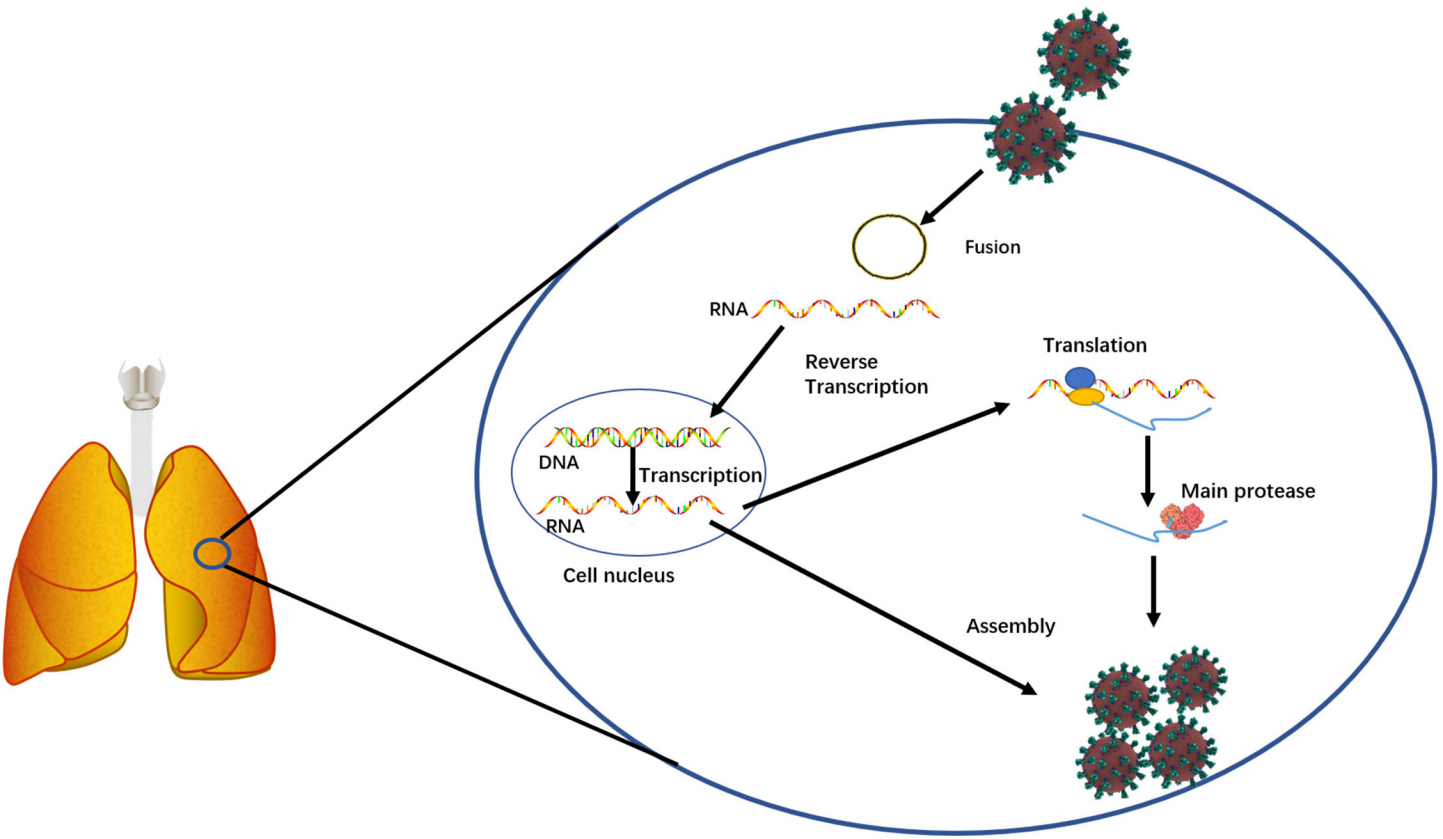
A simplified diagram showing the essential function of the main protease involved in SARS-Cov-2 replication in infected host cells.

SARS-CoV-2 is a novel severe acute respiratory syndrome-related coronavirus strain and highly contagious to humans (Figure 1). Its infection on humans was reported in the early December of 2019 in Wuhan, China (Wang C. et al., 2020; Wu et al., 2020). On January 30, 2020, The World Health Organization (WHO) declared the outbreak of the coronavirus disease 2019 (COVID-19) emergency (World Health Organization, 2020a). On March 11, 2020, WHO announced the COVID-19 pandemic (World Health Organization, 2020b). As of October 22, 2020, it has infected ~41,561,983 people and caused ~1,135,289 deaths worldwide (data source from Johns Hopkins Coronavirus Resource Center). These numbers are continuously increasing every day. Extremely sadly, the humans do not know when this pandemic will end. Although hydroxychloroquine, chloroquine, and remdesivir were recommended for treating COVID-19 (Colson et al., 2020; Sehailia and Chemat, 2020; Wang M. et al., 2020), their therapeutic effects still remain for studies. For example, a new study showed that chloroquine could not treat COVID-19 (Hoffmann et al., 2020). Although 300 trials with different anti-virus medicines are being performed (Sanders et al., 2020), such as lopinavir and ritonavir (Cao et al., 2020), the fact is that no medicines show therapeutic effectiveness. Although in June 2020, dexamethasone, a steroid, was publically reported to decrease the death risk of COVID-19 patients (Khan and Htar, 2020; Selvaraj et al., 2020), more trials need to be performed to demonstrate its effectiveness. Furthermore, different proteins or genes of SARS-Cov-2, such as the main protease (Figure 1), have been targeted to screen medicines, however, no small molecules have been conclusively shown to be able to treat COVID-19 patients (Dai W. et al., 2020; Zhang et al., 2020). Currently, the humans are placing a hope on vaccines. However, no effective vaccines are ready for prevention. The potential risks of vaccines remain largely unknown. Making matters worse, more studies have shown that the originality and the transmission of this contagious virus are more complicated than the humans know. Studies have shown that in addition to aerosol transmission, this virus can be transmitted through gastrointestinal infection (Lamers et al., 2020; Xiao et al., 2020) and can stably stay for 3 h in air and 72 h on plastic and steal surfaces (van Doremalen et al., 2020). In addition to causing lung diseases, this virus has been found to cause other health complications, such as abdominal pain (Lamers et al., 2020) and neurologic abnormality (Helms et al., 2020). To the whole world surprise, one newest study revealed that SARS-Cov-2 existed in wastewater that had been stored in Barcelona, Spain since March 2019 (Chavarria-Miró et al., 2020), the time of which was 9 months earlier than the first report from Wuhan. This finding implies that SARS-Cov-2 might have been transmitted in humans before the outbreak. In summary, no medicines can treat COVID-19 and no vaccines can prevent this contagious disease. Therefore, effective treatments and preventions are urgently needed.

**Figure 2:**
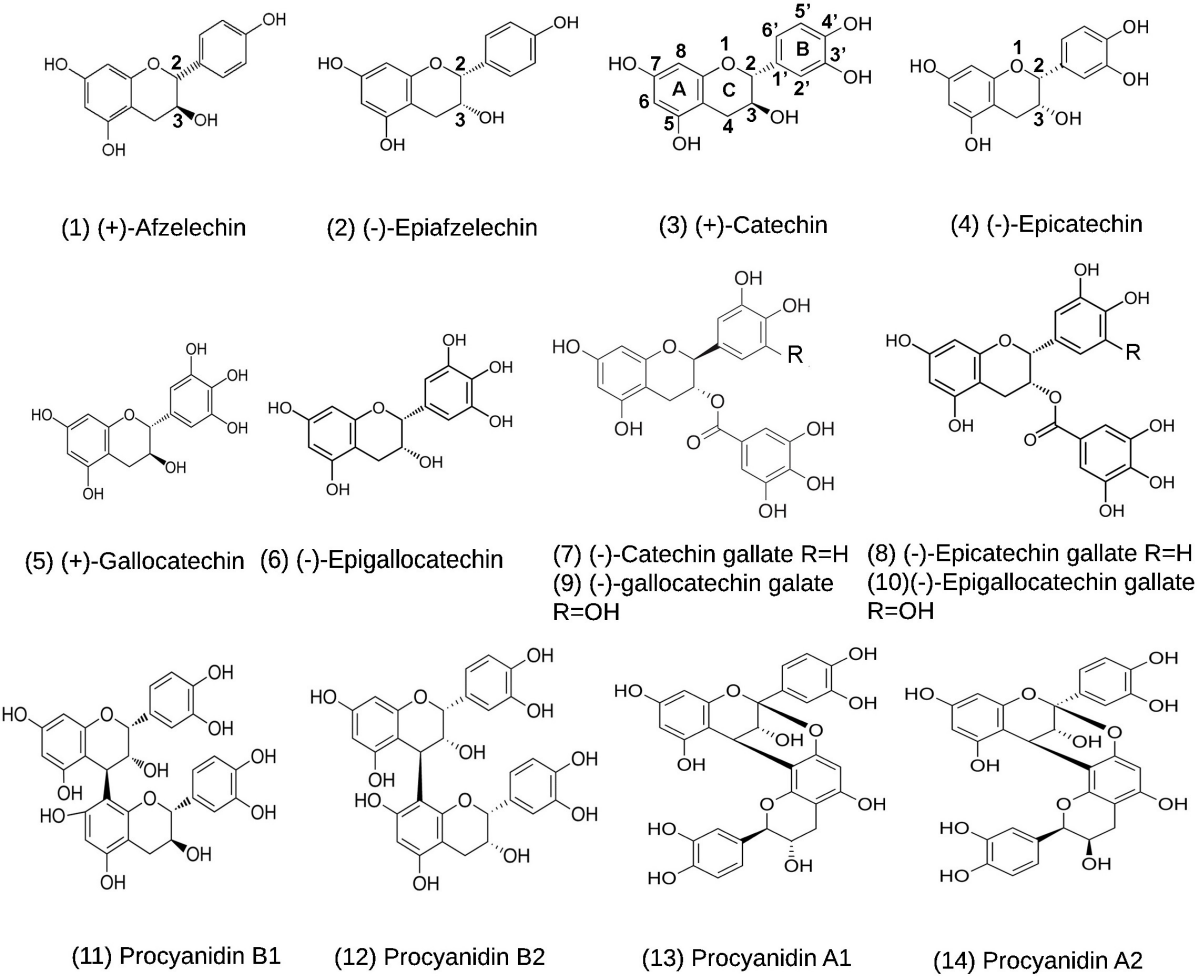
Structures of (+)-afzelechin, (–)-epiafzelechin, (+)-catechin, (–)-epicatechin, (+)-gallocatechin, (–)-epigallocatechin, (–)-catechin-3-O-gallate, (–)-epicatechin-3-O-gallate, (–)-gallocatechin-3-O-gallate, and (–)-epigallocatechin-3-O-gallate, procyanidin B1 and B2, and procyanidin A1 and A2.

Flavan-3-ols and proanthocyanidins (PAs) are two groups of plant flavonoids (Figure 2; Xie and Dixon, 2005). They commonly exist in fruits, food products, and beverages, such as grape (Iacopini et al., 2008; Zhu et al., 2014; Yuzuak et al., 2018; Rousserie et al., 2019), strawberry (Lopez-Serrano and Barcelo, 1997; Fossen et al., 2004; Fischer et al., 2014), persimmon (Akagi et al., 2009), cranberry (Foo et al., 2000a,b), blueberry (Gu et al., 2002), cacao nuts (Murphy et al., 2003; Miller et al., 2009), chocolate (Schewe et al., 2002; Serafini et al., 2003), green tea (Liu et al., 2012; Zhao et al., 2017; Wang P. et al., 2020), and wines (Monagas et al., 2003). Common flavan-3-ol aglycones in these plant products include (–)-epicatechin (EC), (+)-catechin (CA), (–)-epigallocatechin (EGC), (+)-gallocatechin (GC), (–)-epiafzelechin (EAF), and (+)-afzelechin (AF) (Figure 2; Xie and Dixon, 2005). Common flavan-3-ol gallates include (–)-epicatechin-3-O-gallate (ECG), (+)-catechin-3-O-gallate (CAG), (–)-gallatechin-3-O-gallate (GCG), (–)-epigallocatechin-3-O-galloate (EGCG), which are highly abundant in green tea (Dai X. et al., 2020; Wang P. et al., 2020). PAs are oligomeric or polymeric flavan-3-ols. In PAs, the lowest and upper units are termed as the starter and extension units, which are linked by interflavan bonds formed between the C_8_ or C_6_ of a lower unit and the C_4_ of an upper unit (Figure 2). In addition, a second linkage is a C_2_-O-C_7_ ether bond between the starter unit and the extension unit. Based on the linkage numbers, PAs are classified into two types of structures, the dominant B-type characterized with an interflavan bond only and the uncommon A-type featured with an interflavan bond and an ether linkage (Xie and Dixon, 2005). Common dimeric B-types in fruits and beverages include procyanidin B1, B2, B3, and B4. Two examples of dimeric A-type PAs are procyanidin A1 and A2 (Xie and Dixon, 2005). Flavan-3-ols and PAs are potent antioxidants with multiple benefits to human health (Xie and Dixon, 2005). Multiple compounds from these two groups, such as CA, EPC, EGC, EGCG, procyanidin B2, and procyanidin A2 (Figure 2), have been shown to have antiviral function (de Bruyne et al., 1999; Iwasawa et al., 2009), antibacterial activity (Molan et al., 2001; Howell et al., 2005), anticancer (Ohata et al., 2005; Suganuma et al., 2011), anti-cardiovascular diseases (Loke et al., 2008; Panneerselvam et al., 2010; MacRae et al., 2019), and anti-aging diseases (Levites et al., 2003; Li et al., 2004; Weinreb et al., 2004). In particular, the anti-viral activity suggests that flavan-3-ols and PAs are appropriate targets for screening potential anti-SARS-Cov-2 medicines.

In this study, our goal was to use the M^pro^ of SARS-Cov-2 for docking simulation to screen flavan-3-ols and PAs, to identify potential active candidates, and to characterize their binding similarity and difference among various structures. Then, based on positive docking results, we used the M^pro^ of SARS-Cov-2 to perform *in vitro* inhibitory experiments. Five compounds were identified to have anti-M^pro^ activity. Crude extracts from green tea, cacao, chocolate, and two muscadine grapes, which are rich in flavan-3-ols and PAs, also showed inhibitory effects on the M^pro^ activity.

## Materials and Methods

### Candidates of Flavan-3-ols, Flavan-3-ol Gallates, and PAs for Docking Simulation

Main flavan-3-ol aglycones include (+)-afzelechin, (–)-epiafzelechin, (+)-catechin (CA), (–)-epicatechin (EC), (+)-gallocatechin (GC), and (–)-epigallocatechin (EGC). Galloylated flavan-3-ol conjugates include (–)-epicatechin-3-O-gallate (ECG), (–)-catechin-3-O-gallate, (–)-gallocatechin-3-O-gallate (GCG), and (–)-epigallocatechin-3-gallate (EGCG). Dimeric PAs include procyanidin A1, A2, B1, and B2. Fourteen structures are listed in Figure 2. In addition, three known anti-viral medicines, ebselen, cinanserin, and lopinavir, were used as positive controls.

### Plant Materials

Five types of plant products rich in flavan-3-ols and dimeric PAs were used for extraction. “*Mao-Jian*” tea is one type of top green tea (*Camellia sinensis*) products in China. This product is composed of newly leaves (0.8–1.2 cm in length) of early spring sprouts (harvested around April 5 every year), which were harvested in 2019. Ripen muscadine grape berries of FLH 13-11 and FLH 17-66 were collected in 2011 and 2012, ground into powder in liquid nitrogen, freeze dried, and stored in −80°C freezer (Yuzuak et al., 2018). Cacao (*Theobroma cacao*) seed powder and dark chocolate used were obtained from Ecuador in 2019.

### M^pro^ Docking Simulation

**Figure 3:**
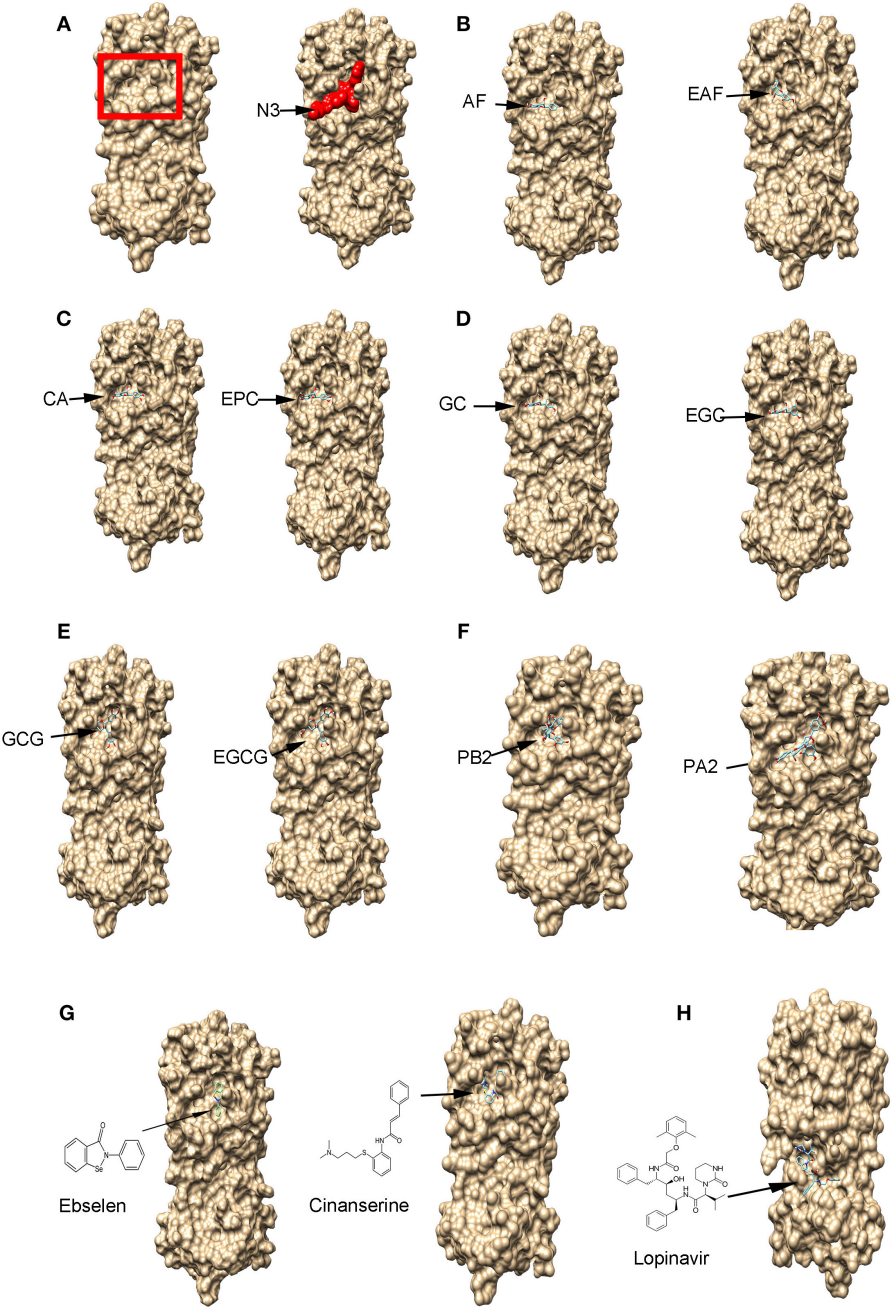
The binding of flavan-3-ols and their gallates, procyanidin B2 and A2, and three potential anti-SARS-Cov-2 drugs to the main protease (M^pro^) shown by protein-ligand docking. **(A)** The 3D surface view of the SARS-Cov-2 M^pro^ without and with the inhibitor peptide N3. The red rectangle frame shows the M^pro^ substrate-binding pocket and the red amino acid residues show the N3 binding to the M^pro^ substrate-binding pocket. **(B–F)** 10 compounds bind to the M^pro^ substrate-binding pocket, (+)-afzelechin (AF) and (–)-epiafzelechin (EAF) **(B)**, (+)-catechin (CA) and (–)-epicatechin (EPC) **(C)**, (+)-gallocatechin (GC) and (–)-epigallocatechin (EGC) **(D)**, (–)-gallocatechin gallate (GCG) and (–)-epigallocatechin gallate (EGCG) **(E)**, and procyanidin B2 and A2 **(F)**. **(G)** Ebselen (EBS) and cinanserin (INN) bind to the M^pro^ substrate-binding pocket. **(H)** Lopinavir (LOP) binds to M^pro^ at a different position.

To understand whether flavan-3-ol aglycones, flavan-3-ol gallates, and dimeric PA molecules (Figure 2) could have anti-SARS-Cov-2 activity, we used M^pro^ to perform docking simulation via two publically available software's websites Dock Prep tool of UCSF-Chimera (https://www.cgl.ucsf.edu/chimera/docs) and AutoDock vina (http://vina.scripps.edu/). In addition, three reported potential anti-COVID-19 candidate compounds, ebselen (Jin et al., 2020a), cinanserin (Chen et al., 2005; Jin et al., 2020a), and lopinavir (Cao et al., 2020), were used as positive controls for docking. We used four steps to complete docking simulation. First, we obtained a SARS-Cov-2 M^pro^ (PDB ID: 6LU7) structure curated in the Protein Data Bank (https://www.rcsb.org/), which consisted of M^pro^ and an inhibitor N3 (Figure 3A; Jin et al., 2020a). Second, we used the Dock Prep tool of UCSF-Chimera to perform receptor (M^pro^) preparation, during which the inhibitor N3 was removed. In addition, hydrogens were added and receptor charge was optimized, which allowed determining the histidine protonation state. The resulting M^pro^ structure file was saved in the mol2 format. Third, the 3D structures of flavan-3-ols and other interesting compounds used in this study were obtained from PubChem (https://pubchem.ncbi.nlm.nih.gov/) and used as ligands. All ligand structures were then uploaded to the Minimize structure tool of UCSF-Chimera and minimized, during which charges and hydrogens were added to each ligand. The resulting ligand files were also saved in the mol2 format. Fourth, both M^pro^ and ligand files were uploaded to AutoDock vina for docking simulation. When we preformed simulation, based on the three dimensional model size of M^pro^, we used a functional tool in the software to create a grid box in a three-dimensional Cartesian coordinate space. The resulting box size was *x* = 50, *y* = 55, and *z* = 50 and the origin center of the box was at *x* = −27, *y* = 13, and *z* = 58. The entire M^pro^ protein was completely framed in the grid box to allow docking simulation at any locations, such as the substrate binding pocket and surface. The advantage of this complete framing was that the simulation excluded potential position bias in the selection of binding sites. Accordingly, different poses of ligands in the same binding sites could be clustered to determine the optimal binding position, which was characterized by the best docking score (affinity score). Moreover, at the optimal position, snapshots were selected to determine the ligand pose and the potential linkage such as hydrogen bond between the ligand and amino acids of M^pro^.

### Extraction of Flavan-3-ols and PAs From Green Tea, Cacao, Dark Chocolate, and Muscadine Berry Powders

One gram of dry powder was suspended in 10 ml acetone: deionized water (70:30) contained in a 50 ml falcon tube. The tube was strongly vortexed for 5 min and then sonicated in a water bath for 10 min at the room temperature, followed by centrifugation at 4,000 rpm for 20 min. The resulting supernatant was pipetted to a new 50 ml tube. The remained pellet was extracted again with the same steps. Two extractions were pooled together to obtain 20 ml in volume, which was reduced to 5.0 ml by removing acetone with a nitrogen gas flow at the room temperature. The tube containing the remained water phase was added 1.0 ml chloroform and then strongly vortexed for 2.0 min, followed by centrifugation at 4,000 rpm for 5 min. The resulting lower chloroform phase containing non-polar compounds was removed. This step was repeated once. The tube that contained the remained water phase including flavan-3-ols and PAs was added 5.0 ml ethyl acetate, strongly vortexed for 3.0 min, and then centrifuged at 4,000 rpm for 5.0 min. The resulting upper ethyl acetate phase containing flavan-3-ols and PAs was transferred to a new 50 ml tube. This ethyl acetate extraction step was repeated two times. The three times of ethyl acetate extractions were pooled together and then dried completely with nitrogen flow at the room temperature. The remained residue was dissolved in DMSO to obtain 50 mg/ml extracts and stored in a −20°C freezer until use.

### *In vitro* Inhibition Assay of M^pro^ Activity and Calculation of IC50 Values

CA, EC, GC, EGC, CAG, GAG, ECG, EGCG, PA2, and PB2 were purchased from Sigma-Aldrich (St. Louis, MO) and dissolved in DMSO to prepare 1.0 mM stock solution. A 3CL Protease (M^pro^) (SARS-CoV-2) Assay Kit (BPS bioscience, https://bpsbioscience.com/) was used to test the inhibitory activity of these 10 compounds. The steps of *in vitro* assay followed the manufacturer's protocol. In brief, each reaction was completed in a 25 μl volume in 96-well plates. Each reaction solution contains 150 ng recombinant M^pro^ (the final concentration in the reaction, 6 ng/ μl), 1.0 mM DDT, 50 μM fluorogenic substrate, and CA, EPC, GC, EGC, CAG, GAG, ECG, EGCG, PA2, PB2 (final concentrations, 0, 0.1, 0.5, 1, 5, 10, 50, 100, 150, and 200 μM) or one plant extract (final concentrations, 0, 1, 10, 100, 1,000 μg/ml) in pH 8.0 50 mM Tris-HCl and 5.0 μM EDTA buffer. GC376 (50 μM) was used as positive control and Tris-HCl-EDTA buffer was used as negative control. The reaction mixtures were incubated for 4.0 h at the room temperature. The fluorescence intensity of each reaction was measured and recorded on a microtiter plate-reading fluorimeter (BioTek's Synergy H4 Plate Reader for detect fluorescent and luminescent signals). The excitation wavelength was 360 nm and the detection emission wavelength was 460 nm. Each concentration of compounds and extracts was tested five times. A mean value was calculated using five replicates. One-way analysis of variance (ANOVA) was performed to evaluate the statistical significance. The *P*-value < 0.05 means significant differences.

After fluorescent values were recorded, based on negative control, the fluorescent intensities of each reaction were converted to percentages, which were used to develop inhibitory effect curves with the Originlab software (https://www.originlab.com/). All data were imported to the Originlab workbook, which had an Analysis Toolbar including a Rank model fitting tool (a non-linear fitting tool). After data were fitted with the non-linear fitting tool, a Dose-Response Curve (DoseRes) function was used to develop non-linear regression curves. The outputs of the non-linear regression included inhibitory effect curves, standard errors, the half maximal inhibitory concentration (IC50) values and a 95% confidence interval (CI), and a range of values including IC5 with a 95% CI.

### Evaluation of Docking Simulation With Receiver Operating Characteristic Curve

Based on actual positive and negative inhibition data obtained from *in vitro* assay, we used receiver operating characteristic (ROC) curve to evaluate the performance confidence of the docking simulation. To develop a ROC curve, we used “1” to stand for positives and “0” to stand for negatives. Accordingly, the sensitivity and 1-specificity false positive values were calculated for each docking score (Supplementary Table 1), and then applied to the OriginLab software to create a ROC plot. Based on the obtained ROC plot, a potential cut off value for affinity score was obtained.

## Results

### Docking Simulation of Compounds Binding to M^pro^

Theoretical compound screening for inhibitors of the SARS-Cov-2 M^pro^ is an effective approach to identify potential candidates that can be used for trials to test their inhibitory activity against SARS-Cov-2 (Xue et al., 2008; Pillaiyar et al., 2016; Dai W. et al., 2020; Jin et al., 2020b; Zhang et al., 2020). To predict whether flavan-3-ol aglycones, their gallates, and dimeric PAs (Figure 2) could inhibit the M^pro^ activity, we performed docking simulation via two types of publically available software, Dock Prep tool of UCSF-Chimera and AutoDock vina. The resulting docking data showed that all flavan-3-ol aglycones, flavan-3-ol gallates, and dimeric procyanidins tested (PA2 and PB2) (Figure 2) could bind to the same location of M^pro^, which is the substrate binding pocket bound by the N3 inhibitor (Figures 3A–F). Given that the docking simulation was completed from any positions, different poses of binding were clustered (Supplementary Figure 1) to determine the optimal position for each ligand (Figures 3A–F). In addition, two antiviral control compounds, ebselen and cinanserin, bound to the same binding pocket (Figure 3G). In contrast, the antiviral lopinavir bound to a different location (Figure 3H).

Based on the optimal pose of ligand (Figures 3B–F), the affinity scores were recorded from the docking simulation. The resulting best affinity scores were −7.0 to −7.7 for six flavan-3-ol aglycones, −8.3 to −8.7 for four flavan-3-ol gallates, and −9.2 for two dimeric proanthocyanidins (Table 1). All these scores were lower than those of ebselen and cinanserin, −6.6 and −5.4. The affinity scores of the dimeric PAs and flavan-3-ol-gallates were lower than −8.0 for lopinavir. These data suggested that flavan-3-ols, flavan-3-ol gallates, and dimeric PAs were appropriate candidates with potentially inhibitory effects on the M^pro^ activity. During our manuscript revision, a recent *in silica* docking for flavan-3-ols in green tea reported similar affinity score ranging from −7.2 to 9.0 (Ghosh et al., 2020). Another *in silica* docking reported the procyanidin B7 and A2 affinity scores, −8.0 and −8.2 (Prasanth et al., 2020). These two studies together with ours suggest that different types of software can give similar simulation data. In addition, potential binding to other sites such as surface was observed in the docking simulation. However, compared with those promising affinity scores (Table 1) obtained from the substrate binding site, the affinity scores from other sites were bigger than −2.0, which were insignificant for further analysis.

**Table 1:** The affinity scores of AF, EAF, CA, EPC, GC, EGC, GCG, EGCG, PA2, and PB2 and three putative anti-COVID-19 molecules binding to the main protease.

**Molecules**	**Affinity score**	**Molecular weight**
	**(kcal/mol)**	**(g/mol)**
Procyanidin B2 (PB2)	−9.2	578.53
Procyanidin A2 (PA2)	−9.2	576.5
(–)-epigallocatechin-3-O-gallate (EGCG)	−8.7	458.37
(–)-gallocatechin-3-O-gallate (GCG)	−8.7	458.37
(–)-epicatechin-3-O-gallate (ECG)	−8.7	442.37
(+)-catechin-3-O-gallate (CAG)	−8.3	442.37
(–)-epigallocatechin (EGC)	−7.7	306.27
(+)-gallocatechin (GC)	−7.6	306.27
(–)-epicatechin (EPC)	−7.5	290.27
(+)-catechin (CA)	−7.5	290.27
(–)-epiafzelechin (EAF)	−7.5	274.26
(–)-afzelechin (AF)	−7.0	274.26
Lopinavir (LOP)	−8.0	628.81
Ebselen (EBS)	−6.6	274.17
Cinanserin (CIN)	−5.4	340.49

### Spatial Binding Characteristics at Different Subsites of M^pro^

**Figure 4:**
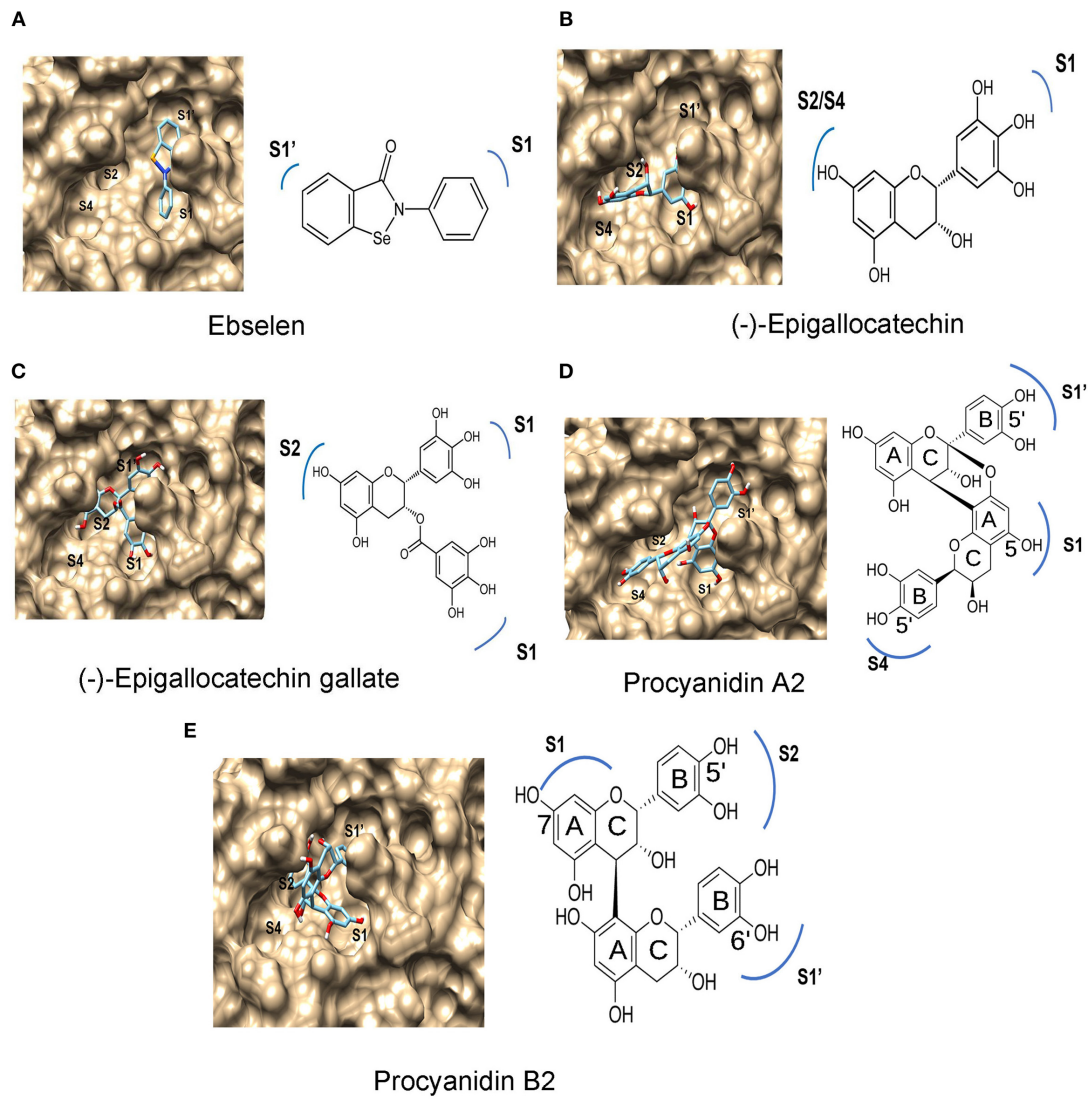
Features of ebselen, (–)-epigallocatechin, (–)-epigallocatechin 3-O-gallate (EGCG), procyanidin A2 (PA2), and procyanidin B2 (PB2) binding to subsites in M^pro^ predicted by protein-ligand docking modeling. **(A)** Ebselen binds to S1 and S1′ subsites. **(B)** (–)-epigallocatechin binds to S1, S2/S4 subsites of the M^pro^. **(C)** (–)-epigallocatechin 3-O-gallate binds to the S1, S1′, and S2 subsites of M^pro^, **(D)** procyanidin A2 binds to the S1′, S1, and S4 subsites of M^pro^. **(E)** Procyanidin B2 binds to the S1, S1′, and S2 subsites of M^pro^.

The substrate binding pocket of M^pro^ has four subsites, S1′, S1, S2, and S4 (Dai W. et al., 2020). Substrate-binding analysis was completed for ebselen (positive control) and 14 compounds (Figure 2). The results predicted different binding features among ebselen, six flavan-3-ol aglycones, four flavan-3-ol-gallates, and two dimeric PAs. The two benzene rings of ebselen were predicted to face to S1 and S1′ (Figure 4A), while the 14 compounds were predicted to face to three or four subsites (Figures 4B–E). The A-ring and B-ring of EGC were predicted to face to S2/S4 and S1, respectively (Figure 4B). The same patterns were predicted for AF, EAF, CA, EPC, and GC. The A-ring, B-ring, and the gallate ester group of EGCG were predicted to face to S2, S1′, and S1, respectively (Figure 4C). The same patterns were obtained for CAG, ECG, and GCG. In the PA1 and PA2 binding, the A-ring and B-ring of the starter unit and the B-ring of the upper unit were predicted to face to S1, S4, and S1′, respectively (Figure 4D). In PB1 and B2 docking modeling, the B-ring of the starter unit and the A-ring and B-ring of upper unit were predicted to face to S1′, S1, and S2, respectively (Figure 4E).

### Potential Hydrogen Bonds Formed Between Compounds and the Binding Pocket of M^pro^

**Figure 5:**
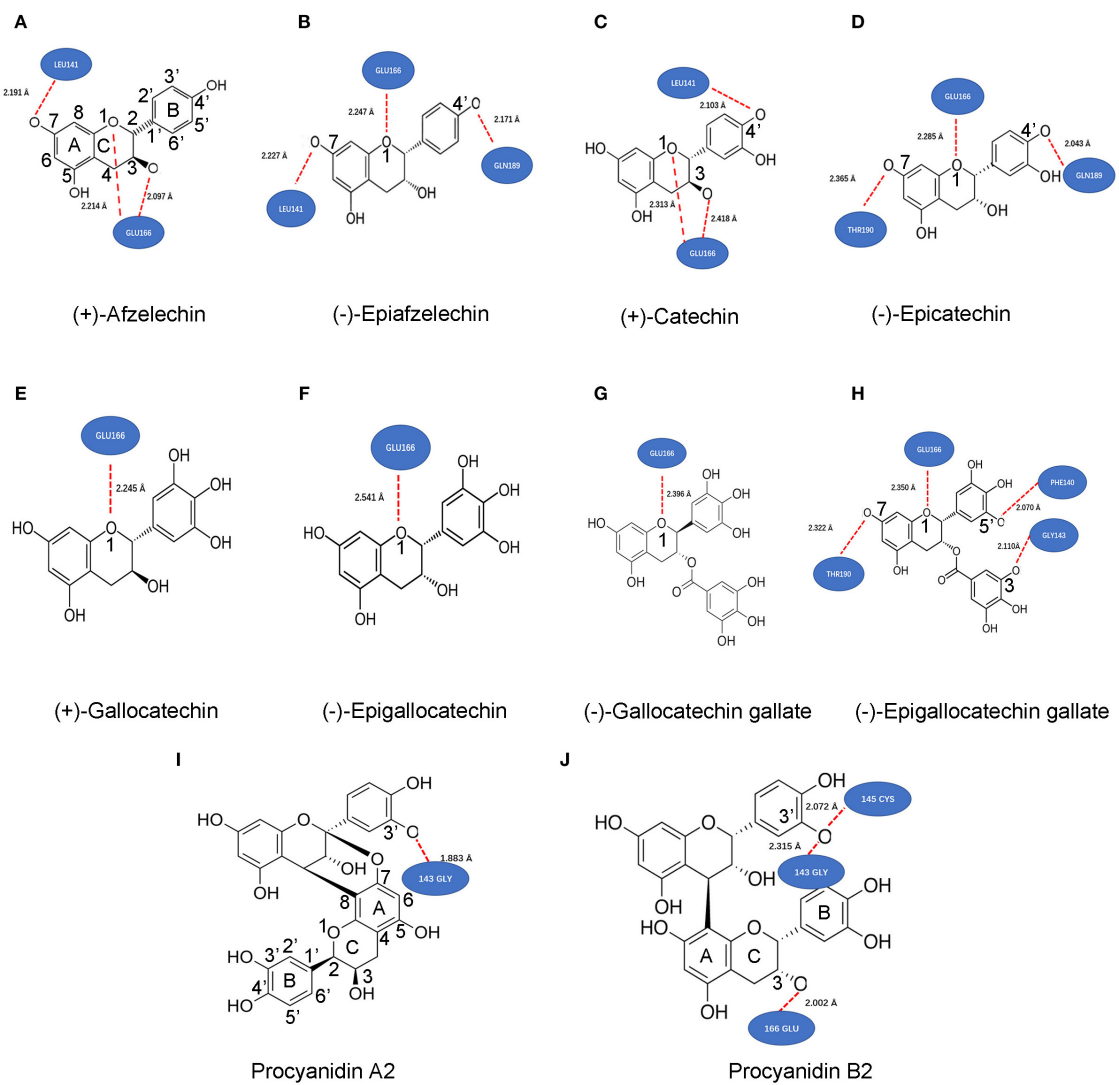
Potential hydrogen bonds formed between M^pro^ and 10 compounds predicted by ligand-protein modeling. These bond positions and numbers formed between 10 various metabolites and the binding pocket of M^pro^ were predicted based on the best affinity scores of each compound (Table 1). (+)-afzelechin (AF) **(A)**, (–)-epiafzelechin **(B)**, (+)-catechin (CA) **(C)**, and (–)-epicatechin **(D)** were predicted form three hydrogen bonds with M^pro^. The hydrogen patterns were the same between AF and CA, while those were the same between EAF and EC. (+)-Gallocatechin **(E)**, (–)-epigallocatechin **(F)**, and (–)-gallocatechin gallate **(G)** were predicted to form one hydrogen with M^pro^. **(H)** (–)-epigallocatechin gallate was predicted to form three hydrogen bonds with M^pro^. Procyanidin A2 **(I)** and B2 **(J)** were predicted to form one and three hydrogen bonds with M^pro^.

This docking simulation predicted that the binding of these compounds to the substrate binding pocket was via the formation of hydrogen bonds. Based on the best affinity score of each compound (Table 1), the docking simulation could predict the most potential hydrogen bonds formed between each compound and the binding pocket of M^pro^. The resulting data showed different features of the hydrogen bond number and potential linkage positions between compounds and amino acids of M^pro^ (Figures 5A–J). Three hydrogen bonds were predicted to be formed between AF, EAF, CA, or EC and M^pro^ (Figures 5A–D). The linkages of those three hydrogen bonds were formed between AF and M^pro^ via O_1_-Glu_166_, C_3_-O-Glu_166_, and C_7_-O-Leu_141_ (Figure 5A), between EAF and M^pro^ via O_1_-Glu_166_, C_7_-O-Leu_141_, and C_4′_-O-Gln_189_ (Figure 5B), between CA and M^pro^ via O_1_-Glu_166_, C_3_-O-Glu_166_, and C_4′_-O-Leu_141_ (Figure 5C), and between EC and M^pro^ via O_1_-Glu_166_, C_7_-O-Thr_190_, and C_4′_-O-Gln_189_ (Figure 5D). GC, EGC, and GCG were predicted to form one hydrogen bond with M^pro^ via O_1_-Glu_166_ (Figures 5E–G). EGCG and M^pro^ were predicted to form four hydrogen bonds via O_1_-Glu_166_, C_7_-O-Thr_190_, C_5′_-O-Phe_140_, and gallate-3-O-Gly_143_ (Figure 5H). PA2 and M^pro^ were predicted to form one hydrogen bond via C_3′_-O-Gly_143_. PB2 and M^pro^ were predicted to form three hydrogen bonds via C_5′_-O-Gly_143_ and C_5′_-O-Cys_145_ on the B ring of the upper unit and C_3_-O-Glu_166_ on the C-ring of the starter unit. Furthermore, all hydrogen-binding distances were different from 1.833Å between PA2 and M^pro^ (Figure 5I) to 2.541 Å between EGC and M^pro^ (Figure 5F).

### *In vitro* Inhibitory Effects of 10 Compounds on the M^pro^ Activity

**Figure 6:**
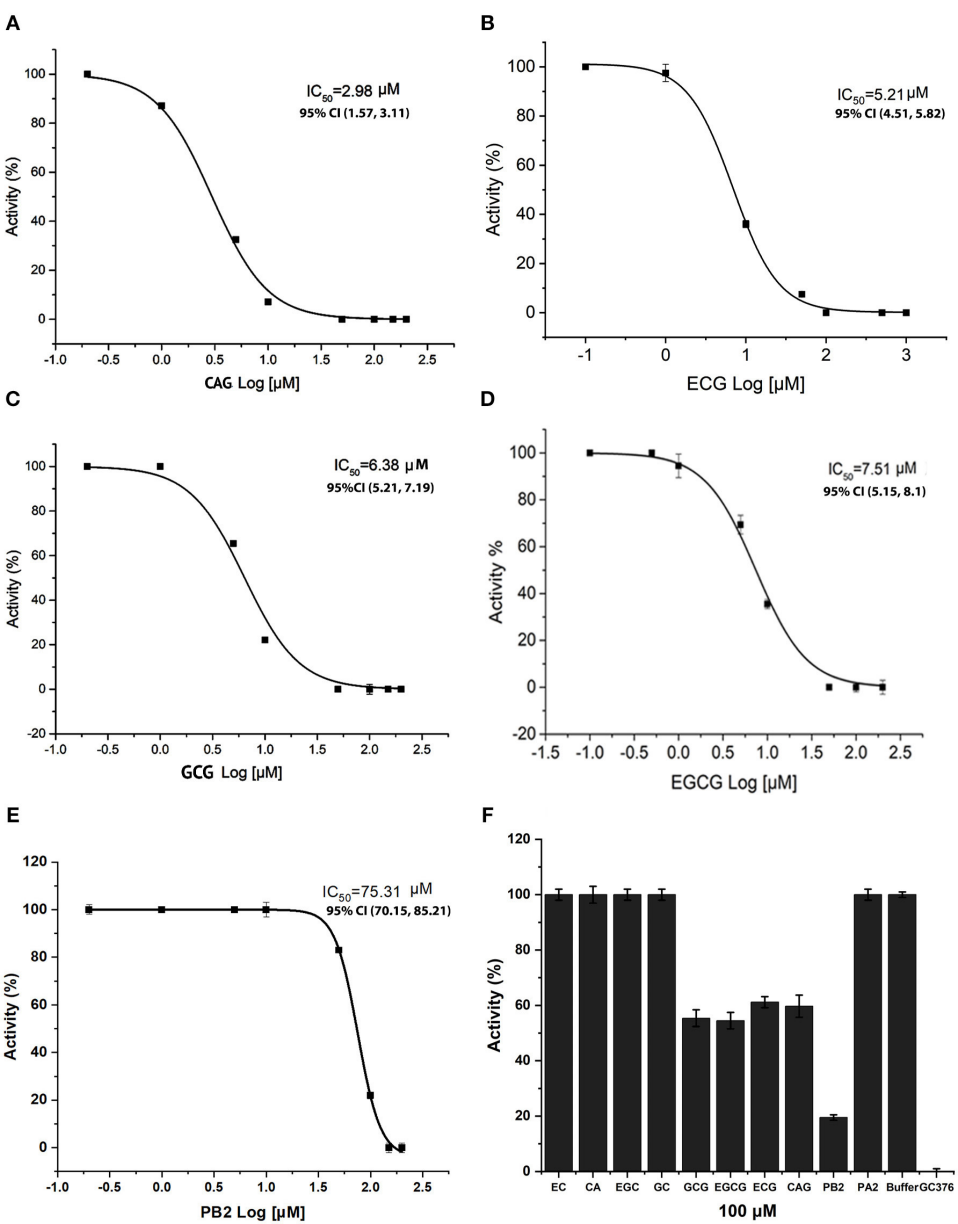
Inhibitory effects of four flavan-3-ol-gallates, four flavan-3-ol aglycones, procyanidin A2 (PA2), and procyanidin B2 (PB2) on the M^pro^ activity of SARS-COv-2. Inhibitory curves show that (+)-catechin-3-O-gallate (CAG) **(A)**, (–)-epicatechin-3-O-gallate (ECG) **(B)**, (–)-gallocatechin-3-O-gallate (GCG) **(C)**, (–)-epigallocatechin-3-O-gallate (EGCG) **(D)**, and PB2 **(E)** inhibit the activity of M^pro^ with an IC_50_ 2.98 ± 0.21 μM, IC_50_ 5.21 ± 0.5 μM, IC_50_ 6.38 ± 0.5 μM, IC_50_ 7.51 ± 0.21 μM, and IC_50_ 75.31 ± 1.29 μM, respectively. **(F)** Inhibitory effects of 100 μM (–)-epicatechin (EC), (+)-catechin (CA), EGC, CAG, ECG, GCG, EGCG, PA2, and PB2 on the activity of M^pro^. Compared with the negative control, the activity of M^pro^ is inhibited by 40, 40, 44.4, 50, 81.5, and 100% by 100 μM CAG, ECG, EGCG, GCG, PB2, and positive control GC376. 95% CI: 95% confidence internal and (value 1, value 2): values in the range with 95% CI.

To understand the effects of these docking promising compounds on the M^pro^ activity, we used CA, EPC, GC, EGC, CAG, ECG, GCG, EGCG, PA2, and PB2 to perform *in vitro* inhibition assay. The resulting inhibitory curves showed that CAG, ECG, GCG, EGCG, and PB2 inhibited the M^pro^ activity (Figure 6). The values of the half maximal inhibitory concentration (IC_50_) for CAG, ECG, GCG, EGCG, and PB2 were ~2.98 ± 0.21, 5.21 ± 0.5, 6.38 ± 0.5, 7.51 ± 0.21, and 75.3 ± 1.29 μM, respectively (Figures 6A–E). The resulting data showed when the concentrations of these compounds were increased, such as 100 μM, the inhibition of PB2 was more effective than that of CAG, GCG, and EGCG (Figure 6F). In contrast, the *in vitro* analysis did not detect that CA, EPC, GC, EGC, and PA2 in a range of concentrations from 0 to 500 μM tested could inhibit the M^pro^ activity (Figure 6F and Supplementary Figures 2A–E).

### Evaluation With Receiver Operating Characteristic Curve

Based on *in vitro* assay, a receiver operating characteristic (ROC) curve was established to evaluate the performance confidence of the docking simulation. *In vitro* inhibition data and affinity scores were used to calculate sensitivity and 1-specificity positive values (Supplementary Table 1), which were used to establish a ROC curve. The resulting plot showed that the area under the ROC (AUROC) was 0.8864 (Supplementary Figure 3), indicating an excellent performance of the docking simulation. The resulting data indicated that the docking scores higher than −7.6 could be used to predict the non-inhibition of flavan-3-ol aglycones against the M^pro^ activity. On other hand, except for PA2, the docking scores lower than −8.3 could be used to predict the inhibition of flavan-3-ol conjugates and dimers against the M^pro^ activity. Accordingly, this ROC evaluation indicated that the docking simulation was appropriate in predicting the inhibitory activity of flavan-3-ols and their conjugates and dimers.

### *In vitro* Inhibitory Effects of Plant Extracts on the M^pro^ Activity

**Figure 7:**
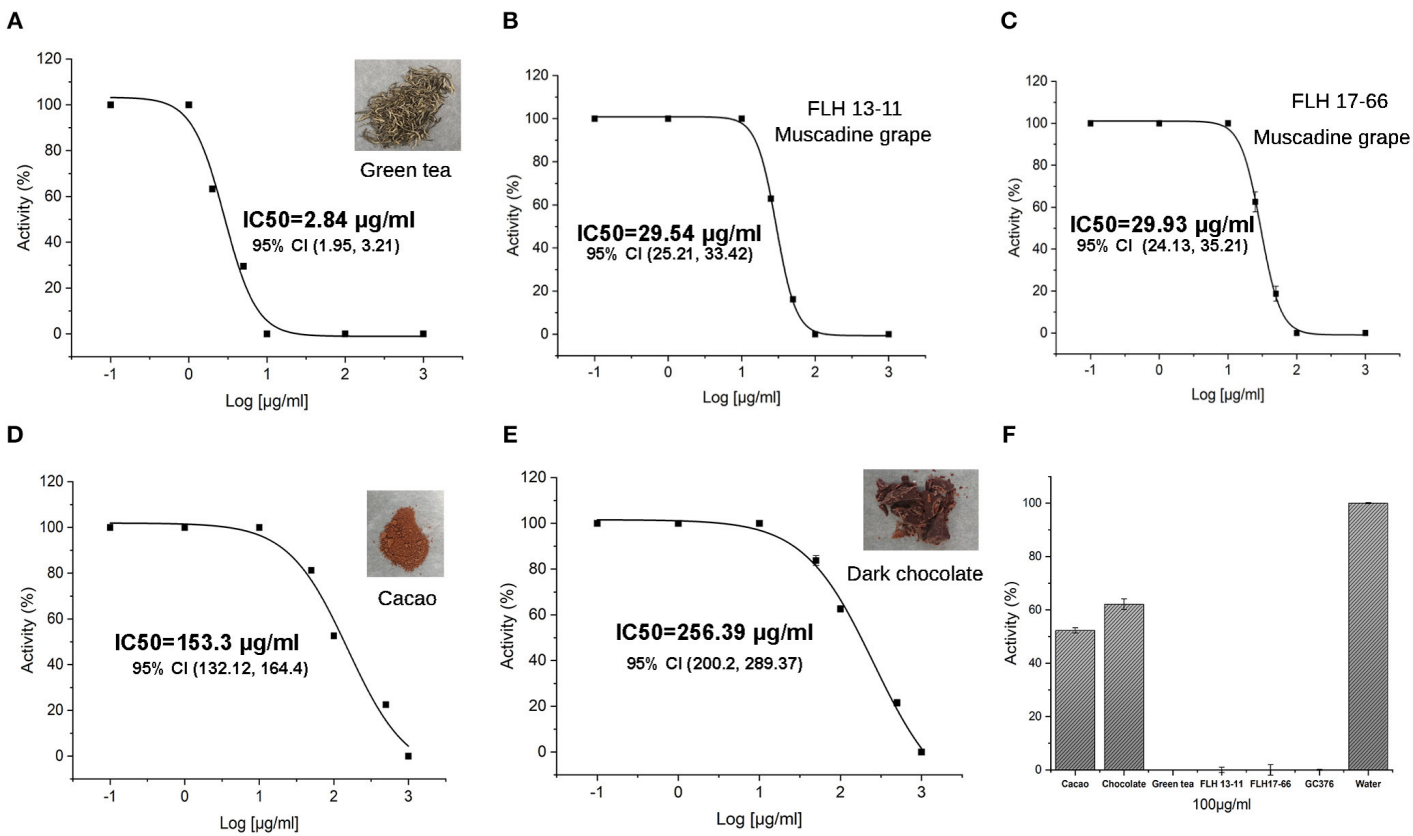
Inhibitory effects of five extracts on the M^pro^ activity. **(A–E)** Curves show inhibitory activities of different concentrations of extracts from green tea with an IC_50_ 2.84 ± 0.25 μg/ml **(A)**, FLH13-11 muscadine berry with an IC50 29.54 ± 0.41 μg/ml **(B)**, FLH17-66 berry with an IC_50_ 29.93 ± 0.83 μg/ml **(C)**, cacao with an IC_50_ 153.3 ± 47.3 μg/ml **(D)**, and dark chocolate with an IC_50_ 256.39 ± 66.3 μg/ml **(E)**. **(F)** Inhibition of the M^pro^ activity by 100 μg/ml extracts of green tea, cacao, chocolate, FLH13-11 muscadine berry, and FLH17-66 muscadine berry. GC376 (100 μg/ml) and 1% DMSO in water were used as positive and negative controls. All values are averaged from five replicates. 95% CI: 95% confidence internal and (value 1, value 2): values in the range with 95% CI.

Green tea (Zhao et al., 2017; Wang P. et al., 2020), two muscadine grape cultivar berries (including seeds and skin) (Yuzuak et al., 2018), and dark chocolate and cacao (Takahashi et al., 1999; Schewe et al., 2001) are rich in flavan-3-ol gallates and dimeric PAs. To understand whether these plant products could inhibit the M^pro^ activity of SARS-Cov-2, we extracted flavan-3-ol gallates and dimeric PAs from the five types of materials and then completed *in vitro* assays. The resulting data showed that all extracts of these products could inhibit the M^pro^ activity (Figures 7A–F). The green tea extracts showed the highest inhibitory activity with an IC_50_ 2.84 ± 0.25 μg/ml. At 10 μg/ml, the green tea extract tea completely inhibited the M^pro^ activity (Figure 7A). In recent, we reported that green tea extracts contained a high content of EGCG, a relatively high content of ECG, and appropriate contents of dimeric procyanidin B1, B2, B3, and B4 (Zhao et al., 2017; Dai X. et al., 2020; Wang P. et al., 2020). Thus, the inhibitory activity of green tea extracts resulted from these compounds. The extracts of two muscadine grapes FLH 13-11 and FLH17-66 inhibited the M^pro^ activity with an IC_50_ 29.54 ± 0.41 μg/ml and an IC_50_ 29.93 ± 0.83 μg/ml. At 100 μg/ml, the extracts of two muscadine grapes completely inhibited the M^pro^ activity (Figures 7B–F). In recent, we also reported that extracts of these two muscadine cultivar berries contained EGCG, ECG, and procyanidin B1-B3 and B5-B8 (Yuzuak et al., 2018). The inhibitory activity of the muscadine extracts was associated with the presence of these compounds. Cacao and dark chocolate extracts were shown to inhibit the M^pro^ activity when the extract concentrations tested were higher than 10 μg/ml and their IC_50_ values were 153.3 ± 47.3 μg/ml and 256.39 ± 66.3 μg/ml, respectively (Figures 7D,E). Given that ECG, EGCG, and PB2 were characterized to be main plant flavonoids in the cacao and dark chocolate extracts (Takahashi et al., 1999; Schewe et al., 2001), the inhibitory activity of these two extracts resulted from these compounds. We further compared the inhibitory activity of all extracts at 100 μg/ml tested, the results showed that the extracts of green tea and two muscadine grapes completely inhibited the M^pro^ activity and the extracts of cacao and dark chocolate reduced the M^pro^ activity by 40–50% (Figure 7F).

## Discussion

Both docking simulation and *in vitro* assays showed that the stereo configurations, galloylation, and oligomeric types of flavan-3-ols affected the ligand-protein binding features and inhibitory activity. Flavan-3-ols and PAs are two groups of plant flavonoids (Xie and Dixon, 2005). PAs are oligomeric or polymeric flavan-3-ols. Flavan-3-ol aglycones have four different stereo configurations at C_2_ and C_3_ on the C-ring (Figure 2). For example, (+)-catechin at C_2_ and C_3_ has a *2R, 3S*-2, 3-*trans* configuration (Figure 2). Its isomer (–)-epicatechin has a *2R, 3R*-2, 3-*cis* configuration. (–)-Catechin and (+)-epicatechin have a *2S, 3R*-2, 3-*trans* and a *2S, 3S*-2, 3-*cis* configuration, respectively (Xie and Dixon, 2005). In addition, the number of hydroxyl groups on the B-ring, such as one on AF, two on CA, and three on GA (Figure 2), diversifies structures. To understand whether these stereo configurations and the hydroxyl group numbers can affect docking simulation and be associated with inhibitory activity, we screened and tested representative flavan-3-ol aglycones, flavan-3-ol gallates, and dimeric PAs. Six flavan-3-ol aglycones included AF, EAF, CA, EC, GC, and EGC (Figure 2), which represented three types of hydroxyl group numbers on the B-ring and two types of stereo configurations at C_2_ and C_3_. All six compounds were predicted to be able to bind to the binding pocket of M^pro^ (Figure 3). It was interesting that the affinity scores decreased as the number of hydroxyl groups on the B-ring of flavan-3-ols increased (Table 1). This datum indicates that more hydroxyl groups on the B-ring increase the binding capacity to M^pro^. Furthermore, the docking simulation showed that the galloylation at C_3_-OH of CA, EC, GC, and EGC and dimerization of EC decreased the affinity scores (Table 1), indicating that the galloylation and oligomerization could increase the binding capacity to M^pro^. Meanwhile, the galloylation of flavan-3-ols increases the acidity of flavan-3-ols and their molecular weights (Table 1). These chemical feature changes might also be associated the increased binding capacity. Based on the docking simulation and affinity scores, we hypothesized that 12 compounds (Figure 2) could have an inhibitory activity against the M^pro^ activity. However, in *vitro* assays only detected the positively inhibitory effects of galloylated flavan-3-ols, including CAG, ECG, GCG, and EGCG, on the M^pro^ activity (Figure 6). No inhibitory activity was detected in the assays of CA, EC, GC, and EGC (Supplementary Figure 2), although their affinity scores were from −7.5 to −7.7. These data indicate that although positive docking data are useful to screen candidates, *in vitro* test experiments are necessary to show an activity. Furthermore, these data showed that the galloylation of these flavan-3-ols created the inhibitory activity against M^°*ro*^. In addition, it was interesting that although the affinity scores of PA2 and PB2 were the same, −9.2, PB2 but not PA2 showed an inhibitory activity against M^°*ro*^ (Figure 6E and Supplementary Figure 2). This datum indicates that the dimeric PA types are closely associated with the inhibitory activities. Based on these *in vitro* assay data and affinity scores, a ROC curve was developed to evaluate the performance confidence of docking simulations for screening flavan-3-ols and PAs (Supplementary Figure 3 and Supplementary Table 1). Although only 12 compounds were screened with docking simulation and 10 compounds were tested *in vitro*, the resulting ROC curve could provide a promising prediction. Based on the ROC curve, the affinity score −8.3 could be proposed to be a cut off value to screen flavan-3-ol derivatives for anti-SARS-Cov-2 candidates.

Although it was difficult to test more flavan-3-ol gallates and dimeric PAs due to the unavailability of compounds, we could take advantage of natural sources to understand the inhibitory effects of more flavan-3-ol gallates and dimeric PAs on the M^pro^ activity. Our previous UPLC-MS based profiling showed that that green tea (GT) produced not only EGCG and PB2 but also ECG, GCG, CAG, procyanidin B1 (PB1), PB3, and PB4 (Zhao et al., 2017; Dai X. et al., 2020; Wang P. et al., 2020). We prepared extracts from GT and tested their activity. The resulting inhibitory activity was effective and the concentration at 10 μg/ml could completely inhibit the M^pro^ activity (Figure 7A). These data suggest that in addition to ECG, GCG, CAG, EGCG, and PB2 tested, PB1, PB3, and PB4 may provide additive inhibitory activity against the M^pro^ activity. In addition, our recent HPLC-MS/MS based profiling reported that the berries (including seeds and peels) of two muscadine grape cultivars, FLH 13-11 FL and FLH 17-66 FL, produced four flavan-3-ol aglycones, 18 galloylated or glycosylated conjugates, and eight dimeric procyanidins (Yuzuak et al., 2018). The extracts of these two muscadine grapes showed an effective inhibitory activity against the M^pro^ activity (Figures 7B,C), indicating that those untested dimeric PAs and flavan-3-ol conjugates might enhance the inhibitory activity. We recently analyzed cacao nut and DW extracts and identified PB1 and PB2 in the extracts. These two extracts also showed appropriately inhibitory activity against the M^pro^ activity. Based on these flavan-3-ol, flavan-3-ol gallate, and dimeric PA-containing natural resources, we hypothesize that those flavan-3-ol gallates, dimeric PAs, and oligomeric PAs untested have inhibitory effects on the M^pro^ activity. Besides, we anticipate that it is valuable to continuously screen more flavan-3-ol gallates and other derivatives as well as PAs, from which effective compounds can be identified for anti-SARS-Cov-2 efforts. In summary, although these natural extracts have not been tested for the inhibitory efficacy in animals and humans, based on their inhibitory activity *in vitro*, we propose that an increased consumption of these common products can enhance preventing against SARS-Cov-2 and improving the COVID-19.

Conclusion, both docking simulation and *in vitro* assay showed that (–)-catechin-3-O-gallate (7), (–)-epicatechin-3-O-gallate (8), (–)-gallocatechin-3-O-gallate (9), and (–)-epigallocatechin-3-O-gallate (10), procyanidin B1 (11) and B2 (12) inhibited the M^pro^ activity of SARS-Cov-2. Moreover, these compound-rich extracts of green tea, muscadine grape, cacao, and dark chocolate also inhibited the M^pro^ activity. Given that there is not an effective medicine for the treatment of COVID-19 and not a vaccine for preventing against the SARS-Cov-2 infection and transmission, these data recommend that these nutraceutical compounds and extracts of green tea, grape, and cacao can be utilized to interfere the devastation of SARS-Cov-2.

## Data Availability Statement

The datasets presented in this study can be found in online repositories. The names of the repository/repositories and accession number(s) can be found in the article/Supplementary Material.

## Author Contributions

D-YX perceived and supervised the entire project, prepared figures, drafted, and finalized the manuscript. YZ completed docking analysis, completed experiments, prepared figures, and drafted materials, methods, and results. Both authors contributed to the article and approved the submitted version.

## Conflict of Interest

The authors declare that the research was conducted in the absence of any commercial or financial relationships that could be construed as a potential conflict of interest.
