# Supplementary material for: Docking Characterization and in vitro Inhibitory Activity of Flavan-3-ols and Dimeric Proanthocyanidins Against the Main Protease Activity of SARS-Cov-2
Source: Front Plant Sci. 2020 Nov 30;11:601316. doi: 10.3389/fpls.2020.601316 (PMC7733993; doi:10.3389/fpls.2020.601316)
Supplement: Supplementary file 1 [file Data_Sheet_1.PDF]

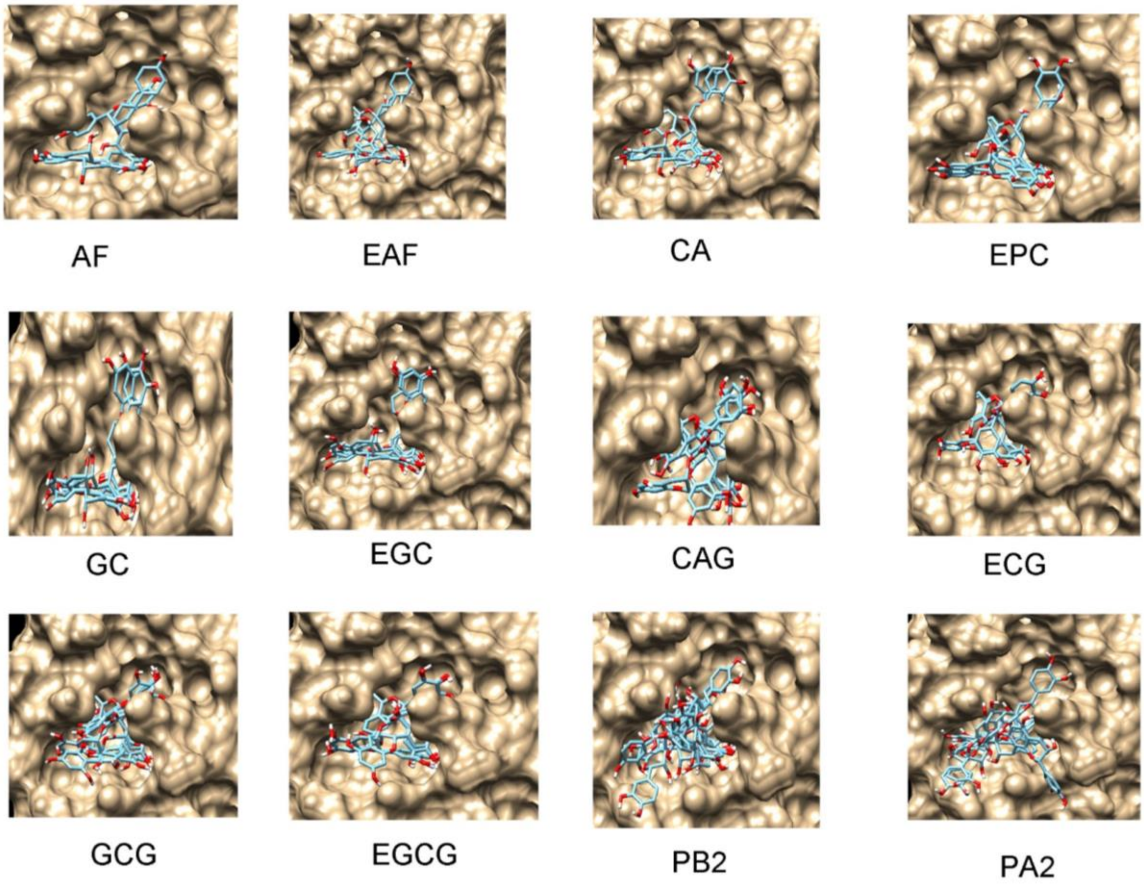

Figure S1 Ligand-receptor docking simulation of flavan-3-ols, PA2, and PB2 against M^{pro} with five different poses overlaid.

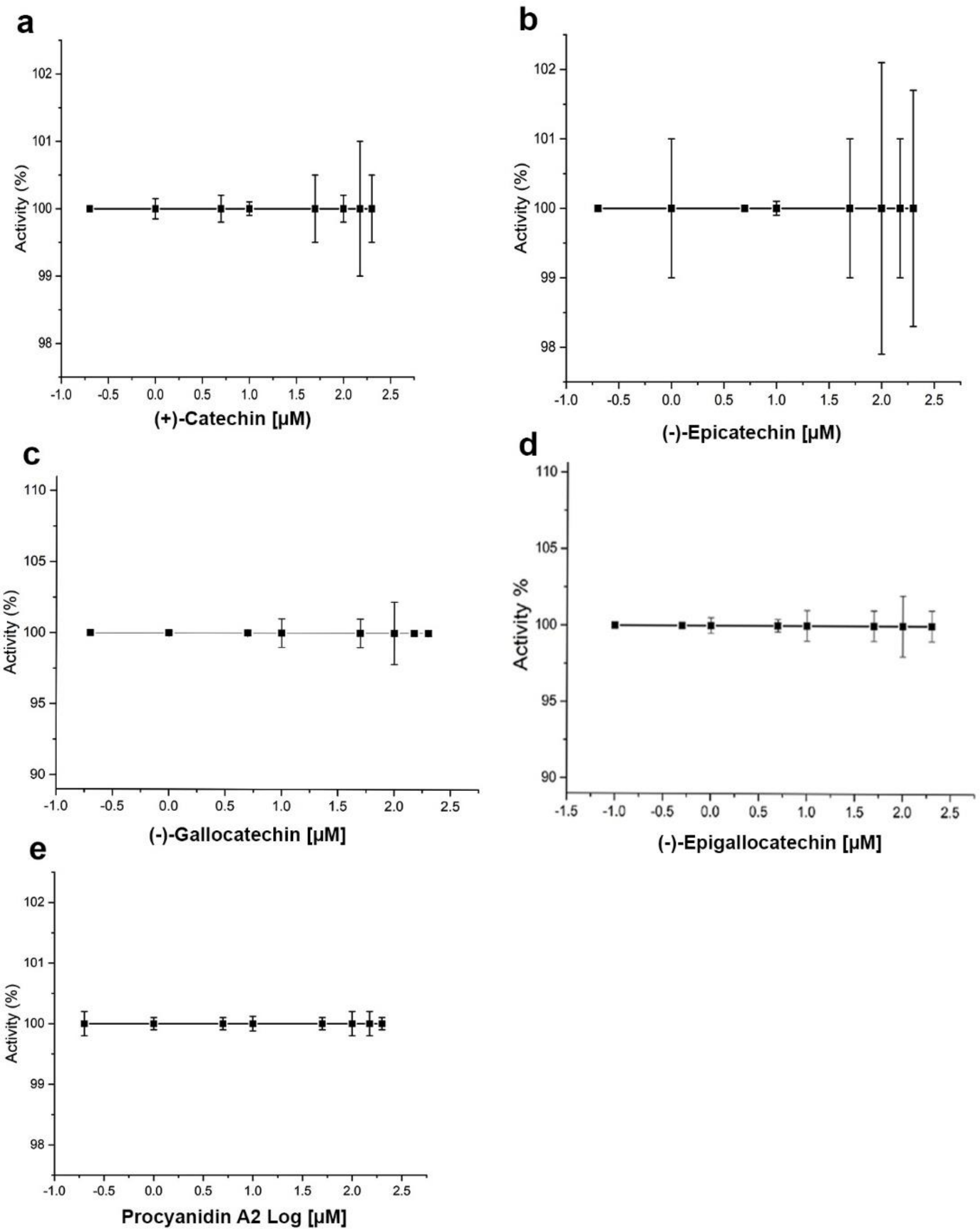

Figure S2 No inhibitory effects of four flavan-3-ols and procyanidin A2 on the activity of M^{pro}. Eight concentrations (0-200 μM) tested for (+)-catechin (a), (-)-epicatechin (b), (-)-gallocatechin (c), (-)-epigallocatechin (d), and procyanidin A2 (e) did not show inhibitory effects on the activity of M^{pro}.

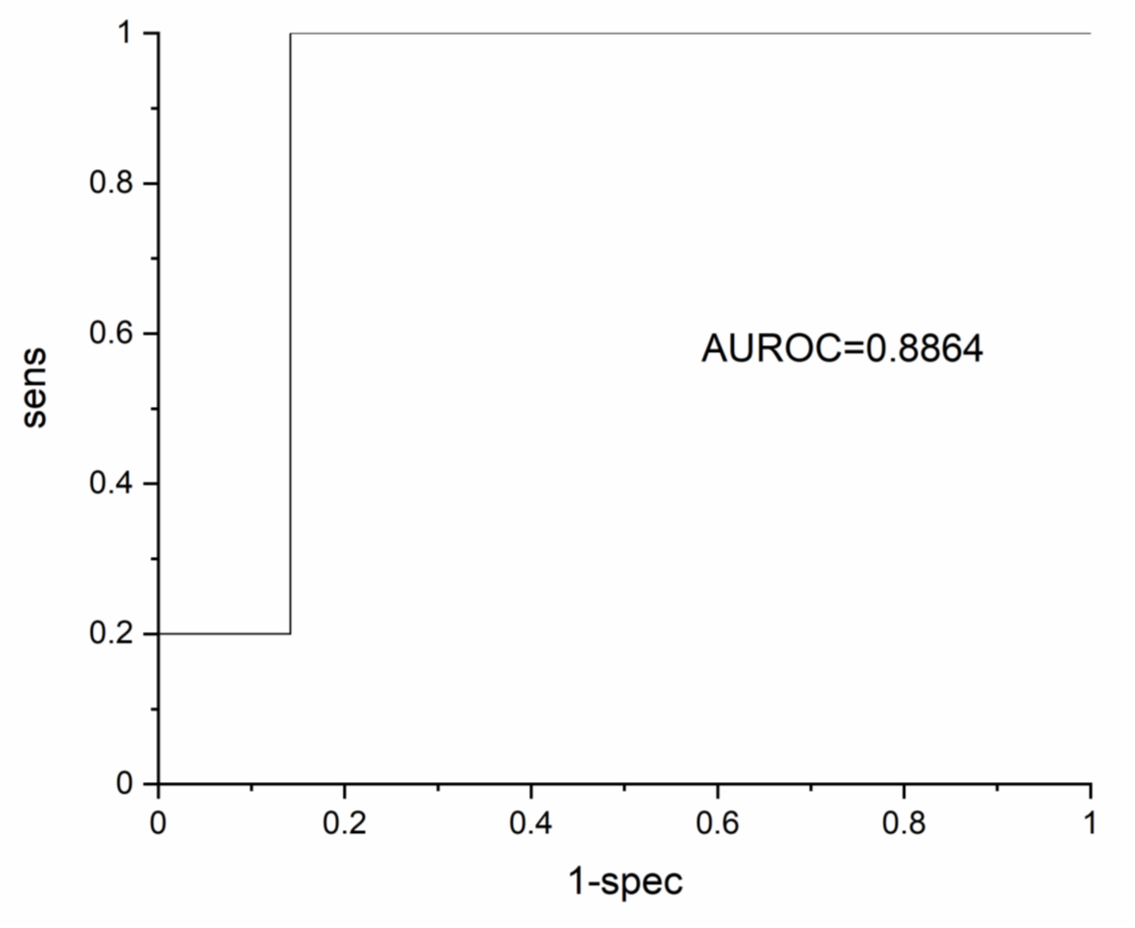

Figure S3 A ROC plot showing the docking simulation performance. The positive and negative inhibition data were assigned as 1 and 0, which were used to calculate sensitivity and 1-specificity false positive values. “sen”: sensitivity; “1-spec”: 1-specificity false positive.

Table S1 Calculation of sensitivity and 1-specificity false positive values for developing a ROC plot to evaluate docking simulation performance and predicting cut off value of docking scores.

Compounds	Docking score	Positive (sum)	Negative (sum)	sensitivity	1-specificity false positive
Procyanidin B2 (PB2)	-9.2	1	0	0.2	0
Procyanidin A2 (PA2)	-9.2	1	1	0.2	0.142
(-)-epigallocatechin-3-O-gallate (EGCG)	-8.7	2	1	0.4	0.142
(-)-gallocatechin-3-O-gallate (GCG)	-8.7	3	1	0.6	0.142
(-)-epicatechin-3-O-gallate (ECG)	-8.7	4	1	0.8	0.142
(+)-catechin-3-O-gallate (CAG)	-8.3	5	1	1	0.142
(-)-epigallocatechin (EGC)	-7.7	5	2	1	0.285
(+)-gallocatechin (GC)	-7.6	5	3	1	0.428
(-)-epicatechin (EPC)	-7.5	5	4	1	0.571
(+)-catechin (CA)	-7.5	5	5	1	0.714
(-)-epiafzelechin (EAF)	-7.5	5	6	1	0.857
(-)-afzelechin (AF)	-7	5	7	1	1
